# The evolving EU regulatory framework for precision breeding

**DOI:** 10.1007/s00122-018-3200-9

**Published:** 2018-10-17

**Authors:** Dennis Eriksson

**Affiliations:** Department of Plant Breeding, Swedish University of Agricultural Sciences, Alnarp, Sweden

## Abstract

Plant breeding has always relied on progress in various scientific disciplines to generate and enable access to genetic variation. Until the 1970s, available techniques generated mostly random genetic alterations that were subject to a selection procedure in the plant material. Recombinant nucleic acid technology, however, started a new era of targeted genetic alterations, or precision breeding, enabling a much more targeted approach to trait management. More recently, developments in genome editing are now providing yet more control by enabling alterations at exact locations in the genome. The potential of recombinant nucleic acid technology fueled discussions about potentially new associated risks and, starting in the late 1980s, biosafety legislation for genetically modified organisms (GMOs) has developed in the European Union. However, the last decade has witnessed a lot of discussions as to whether or not genome editing and other precision breeding techniques should be encompassed by the EU GMO legislation. A recent ruling from the Court of Justice of the European Union indicated that directed mutagenesis techniques should be subject to the provisions of the GMO Directive, essentially putting many precision breeding techniques in the same regulatory basket. This review outlines the evolving EU regulatory framework for GMOs and discusses some potential routes that the EU may take for the regulation of precision breeding.

## The ever expanding plant breeders’ toolbox

Ever since humans started to actively cultivate plants for near-settlement access to harvest 12–14,000 years ago, the genetic make-up of plants has been altered to better suit human needs in terms of cultivation properties and nutritional quality. Starting with the recognition of the Mendelian laws of inheritance early in the twentieth century (Fischer 1936), this breeding process went from a relatively simple on-farm mass selection procedure to an active off-farm scientific procedure whereby the variation in the genetic material is actively increased and suitable genetic combinations are selected for in a much more targeted manner (Smykal et al. 2016). The early stages of scientifically guided plant breeding involved deliberate crosses between suitable parent lines. Later discoveries in physics and chemistry enabled the use of mutagenic agents, such as radiation or chemicals, to induce random mutations followed by a selection procedure of lines with beneficial traits (Ahloowalia and Maluszynski 2001). Later progress in plant cell and tissue culture also allowed wide crosses through protoplast fusion (Glimelius 1988), faster hybrid breeding through anther culture and double haploid induction (Germaná 2011), and yield improvements and wide crosses through polyploidization (Sattler et al. 2016). Progress in molecular biology in the 1960s and 1970s enabled researchers to clone and recombine nucleic acid sequences in a highly specific manner, and the transfer and incorporation into the genome of novel host organisms (Barton et al. 1983; Herrera-Estrella et al. 1983) also paved the way for highly controlled and efficient trait management. This progress was quickly picked up by the commercial seed sector and the first genetically modified (GM) crops were commercialized in the early 1990s. More recent developments in genome editing (Barabaschi et al. 2016) have now also enabled mutagenesis to be carried out in a targeted way, in contrast to the earlier random mutagenesis methods. In other words, modern plant breeding as carried out over the past 120 years is always building on scientific progress in order to expand the available toolbox and provide for a more efficient and targeted trait management. One striking feature though of the molecular breeding is that the genetic alterations, and by extension trait management, became much more targeted and specific. Whereas earlier breeding techniques mostly produced random genetic alterations, molecular precision breeding allows, whenever sequence information is available, specific genes to be pinpointed and modified (Moose and Mumm 2008).

## EU GMO legislation 1.0: early risk management

The products of plant breeding were still in the 1980s by and large not subject to any specific requirements for risk assessment. However, the power and utility of recombinant nucleic acid technology made scientists voice concerns that these versatile techniques could have a potential to be biologically hazardous (Rogers 1975), though it was emphasized that the concerns were based on judgments of potential rather than demonstrated risk (Berg et al. 1974). When the first transgenic plants were then produced in the early 1980s (Barton et al. 1983; Herrera-Estrella et al. 1983), further discussions were triggered about biosafety assessments and regulation of the commercial activities within the field of plant genetic engineering, and the first biosafety legislation on genetically modified organisms (GMOs) was drafted in the European Union (EU) in the late 1980s. This resulted in the first Council Directive on the deliberate release into the environment of GMOs (Directive 90/220/EEC) in 1990 (Official Journal of the European Communities 1990). The ensuing procedure for risk assessment and risk management of GMOs in the EU has thus been established with the purpose of ensuring a high level of protection of human health and the environment. The original intentions when drafting this legislation was to allow for an acknowledgment of the potential benefits of the GM techniques, acknowledge their prospective history of safe use, allow a periodic updating of annexes to accommodate scientific progress, and to progressively shift the focus from the techniques to the resulting organisms and their traits (Commission of the European Communities 1988; Eriksson 2018a). However, these original intentions did not find their way into the Directive 90/220/EC, and have yet to materialize into the implementation of the current EU GMO legislation.

## EU GMO legislation 2.0: centralization and amplification

During the 1990s, a number of food crises affected Europe in parallel with ethical discussions surrounding the emergence of mammal cloning technology. The first GM crops and their derived products had reached the market in Europe, but also the first studies reporting potential health risks with the consumption of GM food products appeared (Ewen and Pusztai 1999). Voices were therefore raised for a higher level of scrutiny and risk assessment of GMOs, and a de facto moratorium on the authorizations of novel GMOs and their derived products was put into effect in the EU in 1998. A process started to reform the GMO legislation, introducing labeling and post-authorization monitoring requirements (Official Journal of the European Union 2003b). Most importantly, the entire risk assessment and authorization procedure was centralized, whereas Directive 90/220/EC had left both the risk assessment and the authorization decision in the hands of the individual EU member states [though other member states had the right to object prior to the actual authorization and, in case of dispute, the final decision was left to the European Commission (EC)]. The European Food Safety Authority was created to provide for a centralized risk assessment and the decision on adoption or rejection was henceforth taken collectively by all member states (or ultimately by the European Commission, EC) (Official Journal of the European Communities 2001; *ibid*, 2002; Official Journal of the European Union 2003a). A number of other details were also introduced, such as a limit to the placing on the market to a period of ten years after which renewal is required, a public consultation before authorization, and in particular a much stronger emphasis on the precautionary principle. Regarding the data required for the risk assessment, these are not specified in Directive 2001/18/EC or Regulation 1829/2003, but over the years EFSA has published an increasing number of guidelines for this purpose. It is now argued that this increasing number of EFSA guidelines is creating an unpredictable situation for the applicants, given that the handling times are also getting progressively longer (EuropaBio 2015). For an excellent and detailed description of how the GMO risk assessment in the EU has developed over the years, it is recommended to read Casacuberta et al. (2017). One problem though with the collective decision-making procedure is that the required qualified majority is almost never reached (Smart et al. 2015; Casacuberta et al. 2017).

## Emerging techniques and the need for a new approach

Since the revised regulatory framework for GMOs was developed in the EU in the early 2000s, there has been considerable progress both in the development of breeding techniques and in our understanding of how naturally dynamic the genetic material is. Over the past decade, there has therefore been considerable debate on what exactly constitutes a GMO within the EU legislative framework, and thus to which organisms the provisions of the Directive applies. The definition in Directive 2001/18/EC (Article 2(2)) says that it is “an organism in which the genetic material has been altered in a way that does not occur naturally by mating and/or natural recombination”. Annex 1A also says that “Techniques of genetic modification referred to in Article 2(2)(a) are *inter alia*: (1) recombinant nucleic acid techniques involving the formation of new combinations of genetic material” (Official Journal of the European Communities 2001). It has therefore been argued that the trigger for the EU regulation is based on the presence of foreign recombinant nucleic acids in the final product (Voytas and Gao 2014; Jones 2015). Other experts have also argued that a combination of process and product is necessary to define a GMO in the EU, given that a number of techniques are specified that do or do not lead to GMOs, while at the same time the product needs to contain recombinant nucleic acids to be a GMO, implying that only “artificially modified” products containing recombinant nucleic acids are encompassed by the regulations (Sprink et al. 2016; Kahrmann et al. 2017). This has been challenged by several green NGOs, who argue that the mere use of a certain technique is sufficient to have the resulting product regulated as a GMO (Friends of the Earth 2016; Greenpeace 2016). The position taken by several EU member states, at least as judged from published position papers and other material, tend to be that a type of recombinant, foreign nucleic acid needs to be present in the final product for it to be regulated as a GMO (for a list of member state opinions and references, see Eriksson 2018b).

A recent ruling from the Court of Justice of the European Union (CJEU) on the legal status of mutagenesis (case C-528/16) gives an indication regarding how the EU GMO legal framework may be applied henceforth. This Court ruling stated that the risks linked to the use of new mutagenesis techniques (i.e. directed mutagenesis) might prove to be similar to those that result from the production and release of a transgenic GMO. Excluding these organisms from the scope of the GMO Directive would therefore compromise the objective, which is to avoid adverse effects on human health and the environment, and the exemption that applies to the products of conventional (randomly induced) mutagenesis should not apply to the products of directed and other new mutagenesis techniques (CJEU 2018). This article does not discuss the merits of the Court ruling on case C-528/16; however, the implications of the ruling will be discussed through a few examples below.

The system has earlier been put to the test on a few occasions. The US-based company Cibus has developed an herbicide-tolerant oilseed rape, using an oligonucleotide-directed mutagenesis approach, that has been declared outside the scope of regulation in the USA and Canada. The company asked six EU member states for the regulatory status of this event, and all member states confirmed that it should not be regulated as GMO. However, the European Commission (EC) instructed the member states to not take any interim national decisions, while there is still regulatory uncertainty at the EU level. Now with the recent ruling on mutagenesis from the Court of Justice of the European Union (CJEU), it seems indeed likely that the EU will regulate the Cibus ODM-oilseed rape as a GMO. Swedish and Finnish research groups have also asked their respective National Competent Authorities (NCAs) for advice on whether or not specific GMO permit is needed for field trials with CRISPR-modified Arabidopsis plants, to which the NCAs in both countries have responded that plants that do not contain foreign DNA will not be regulated as GMO. Again, however, it is unlikely that this approach will be viable in the light of the CJEU mutagenesis ruling. For a more detailed description of the oilseed rape and Arabidopsis cases, see Eriksson (2018a, b).

## Options to regulate the products of plant breeding

Should molecular precision breeding be treated differently compared to breeding techniques that induce mostly random genetic alterations, in terms of requirements for human and/or environmental risk assessment? There is already an indication from the interpretation of mutagenesis that the CJEU delivered on July 25, 2018. The interpretation implied that the products of directed mutagenesis are considered sufficiently distinct from the products of randomly induced mutagenesis to subject them to provisions of the Directive 2001/18/EC, including a requirement for pre-authorization risk assessment and an authorization procedure, whereas the products of randomly induced mutagenesis are exempt from the same. As it is the task of the CJEU only to interpret existing legislation, it is problematic that the EU GMO legislation apparently allows a reasoning that divides the products of mutagenesis into two distinct categories in this way (random vs directed). From a general biosafety perspective, it is difficult to envisage that products carrying a small amount of precisely induced mutations in pre-defined locations (with the possibility of few off-target alterations) should be more risky than products carrying a large amount of random mutations (with a large amount of off-target alterations).

The first obvious question is therefore if this categorization of breeding techniques into “precision” and “conventional” (or “random”) is relevant or necessary? If the answer is no, then the following question is whether the products of conventional breeding should be more strictly risk regulated, or if the products of precision breeding should be less strictly risk regulated? Alternatively, if the best option would be to find a middle way? For the reader who wants to know more about the off-target effects of different breeding techniques, it is recommended to read Filipecki and Malepszy (2006), Schouten and Jacobsen (2007), Batista et al. (2008), Zhou et al. (2012), Schnell et al. (2015), Nelson and Gersbach (2016), Anderson et al. (2016), Batista et al. (2017) and Herman et al. (2017).

Several models have been proposed for the regulation of products resulting from a variation of breeding techniques. The Stanford model suggests a trait-based and technique-independent model where products would be placed into different risk categories on a case-by-case basis (Barton et al. 1997; Miller 2010; Conko et al. 2016). A recently proposed regulatory framework for genome-edited crops suggests that five steps should represent the primary guiding principles: (1) unintentional escape, (2) absence of foreign sequences, (3) documentation of DNA sequence changes at the target site, (4) unintended secondary editing events, and (5) documentation of the above four points (Huang et al. 2016). A much stronger focus on the modified traits, rather than the technique used, has also been suggested (Marchant and Stevens 2015; Ricroch et al. 2016). The Norwegian Biotechnology Advisory Board has also recently initiated discussions on what should be regulated by the Norwegian Gene Technology Act, and proposed an approval system where a three-level hierarchy based on genetic change determines the amount of assessment needed (Norwegian Biotechnology Advisory Board 2018).

## Conclusion

From the way in which the EU GMO legislation was set up and has developed over the years, it is difficult to avoid the general conclusion that in the EU products with highly specific, targeted and pre-defined, and few off-target, genetic alterations (“precision breeding”) are subject to strict, expensive and time-consuming human health and environmental risk assessments and an ensuing authorization procedure, whereas products with randomly induced, and plenty of off-target, genetic alterations (“conventional breeding”) are not subject to these specific risk assessments or the authorization procedure. The above described alternative regulatory models are mostly based on the relevance of technique versus trait, and/or the level (amount) of genetic alteration, and to some extent the origin of transferred genetic material. To my knowledge though, it has not been discussed in the scientific or gray literature whether or not the specificity/precision versus randomness of the genetic alterations would be a relevant factor to consider when setting up the requirements for risk assessment of the resulting products of the breeding process. This is surprising, given that it seems (as shown here above) to be of such prime importance judging from how the EU GMO regulatory framework has evolved, not the least with the recent CJEU ruling on mutagenesis. I therefore suggest that the relevance (or non-relevance) of this factor should be more thoroughly researched and discussed, both from a scientific and from a legal perspective.

### Author contribution statement

DE prepared the initial draft, developed and finalised the manuscript.
